# Wuhan Ionospheric Oblique Backscattering Sounding System and Its Applications—A Review

**DOI:** 10.3390/s17061430

**Published:** 2017-06-18

**Authors:** Shuzhu Shi, Guobin Yang, Chunhua Jiang, Yuannong Zhang, Zhengyu Zhao

**Affiliations:** 1 School of Remote Sensing and Information Engineering, Wuhan University, Wuhan 430079, China; shishuzhu@whu.edu.cn; 2 School of Electronic Information, Wuhan University, Wuhan 430072, China; chuajiang@whu.edu.cn (C.J.); ynzhang@whu.edu.cn (Y.Z.); zhaozy@whu.edu.cn (Z.Z.)

**Keywords:** aircraft detection, high-frequency radar, ionospheric sounding, ocean sensing, single site location

## Abstract

For decades, high-frequency (HF) radar has played an important role in sensing the Earth’s environment. Advances in radar technology are providing opportunities to significantly improve the performance of HF radar, and to introduce more applications. This paper presents a low-power, small-size, and multifunctional HF radar developed by the Ionospheric Laboratory of Wuhan University, referred to as the Wuhan Ionospheric Oblique Backscattering Sounding System (WIOBSS). Progress in the development of this radar is described in detail, including the basic principles of operation, the system configuration, the sounding waveforms, and the signal and data processing methods. Furthermore, its various remote sensing applications are briefly reviewed to show the good performance of this radar. Finally, some suggested solutions are given for further improvement of its performance.

## 1. Introduction

Since high-frequency (HF) radio waves with frequencies between 3 and 30 MHz can be reflected by the ionosphere and propagated by the sea surface, they are not only one of the best candidates for long-range communication [1], but are also an important frequency band for radar to sense the Earth’s environment [2]. Various HF radars have been proposed for military and commercial applications. The first type is the HF over-the-horizon radar (OTHR) that is based on the skywave or ground-wave propagation mode. The skywave OTHR uses the ionosphere to reflect its waves back to the Earth, and can provide radar coverage at distances of about 100–3500 km to monitor aircraft [3], missile launches [4], nuclear explosions [5], ships [6], ocean-surface wind fields [7], cyclones [8], ocean wave height [9], ocean currents [10], and so on. On the other hand, the ground-wave OTHR makes use of the ground propagation, and can detect the targets beyond the horizon to distances of hundreds of kilometers. Considering the practical radar resolution that depends on the width of the antenna beam and the range to the target, this OTHR is most useful for providing early warnings of low-flying aircraft [11], ships [12], sea-skimming missiles [13], and icebergs [14]. It has also already been successfully applied to the remote sensing of surface currents [15], ocean-surface wind fields [16], and the significant waveheight [17]. Another kind of HF radar is the ionosonde, including the oblique backscatter sounder [18], the vertical- and oblique-incidence sounders [19,20,21,22,23], and the HF Doppler sounder [24,25]. These sounders transmit and receive HF radio waves to and from the ionosphere to provide a large amount of information on the behavior and physics of the ionosphere, and they have been widely used for validating ionospheric models [26], studying the characteristics of the ionosphere [27,28,29,30], forecasting and updating the HF channel parameters in real-time [31], studying the long-term influence of the ionosphere on HF radio communication [32], supporting the frequency management system of the skywave OTHR [33], tracking earthquakes [34], observing meteors [35] and the mesospheric winds [36], and so on. Due to the long sounding range, the large path losses, the strong interference in the HF band, and the small radar cross-section (RCS) of some particular targets, high transmit power is usually required in HF radars (especially for the OTHR and the oblique backscatter sounder [37,38]), resulting in a high operation cost and electromagnetic environment pollution. Moreover, most of the traditional HF radar designs are based upon analogue concepts [39]. As a result, the hardware size of these HF radars is always very large, and the flexibility and multifunctionality are not desirable. In recent years, advances in radar technology have provided opportunities to improve the performance of HF radar significantly [40,41,42], and to enable more applications to be tackled [43].

As early as the 1990s, the Ionospheric Laboratory of Wuhan University began to develop a low-power and small-size HF backscatter radar to survey a large region of the ionosphere in real-time, and this radar is referred to as the Wuhan Ionospheric Oblique Backscattering Sounding System (WIOBSS). Pseudorandom coded long-pulse modulation and the long-time coherent spectral integration techniques have been adopted in WIOBSS to obtain additional signal processing gain and to reduce its transmit power. At the same time, integrated circuit chip, digital signal processing, and virtual instrument bus techniques have been used to reduce its dimension. Furthermore, a high degree of flexibility for multifrequency and multimode operations has been offered by the application of modular design, software radar, and programmable signal processing. Hence, this radar can not only operate at HF, but can also operate at very high frequency (VHF) to perform new sensing tasks, such as the detection of natural ionospheric irregularities. With proper time and frequency synchronization schemes, WIOBSS can not only be configured as a multistatic radar to detect a target in a much larger surveillance area and to extract more information from the target, but can also be configured as an HF passive radar to receive the signals emitted by existing HF broadcasts and to perform single site location (SSL). With this new system design, in addition to oblique backscatter sounding, other functionalities have also been achieved with WIOBSS, including the vertical- and oblique-incidence soundings, the passive sounding in the HF band, aircraft detection, and ocean sensing. 

In order to provide some reference solutions for designing a low-power, small-size, and multifunctional radar with high flexibility, progress in the development of WIOBSS is described in this article. Furthermore, a brief review of its various remote sensing applications is given to demonstrate the good performance of WIOBSS. Finally, according to the current problems existing in this radar, several recommendations are provided to further improve its performance.

## 2. Development of WIOBSS Operating at Low Transmit Power

As an HF backscatter radar, on the one hand, basic principles of operation that are based on the characteristics of the ionosphere have been proposed for WIOBSS. On the other hand, advanced radar technology has been adopted in WIOBSS to improve its performance and to enhance its flexibility and applications. The basic principles of operation, the system configuration, the sounding waveforms, and the signal and data processing methods are introduced as follows to gain a better understanding of this radar.

### 2.1. Basic Principles of Operation

The ionospheric channel varies temporally and spatially, and therefore seriously affects the transmission of HF radio waves. As for WIOBSS with low transmit power, the ionospheric channel can be viewed as a linear time-variant system, and its characteristics can be described by the bitemporal response function $h(t,t_{p})$ and the channel scattering function $D(f_{d},t_{p})$, where t, $t_{p}$, and $f_{d}$ denote the time, the group propagation delay, and the Doppler frequency, respectively. As demonstrated by Chong et al. [44] in 2000, the ionospheric channel is stationary within several minutes in most cases. If a pseudorandom sequence whose autocorrelation function has a Dirac shape is used to modulate the phase of the transmitted signal in WIOBSS (e.g., the Huffman code), $h(t,t_{p})$ can be directly achieved for one sounding at a fixed frequency. After multiple soundings at this frequency and a fast Fourier transform of $h(t,t_{p})$, $D(f_{d},t_{p})$ can be obtained. Furthermore, by sweeping over the frequency band of interest with a fixed frequency step, the oblique backscatter ionogram (the group distance versus the operating frequency with color-coded echo amplitudes) and the oblique backscatter Doppler ionogram (the group distance versus the operating frequency with a color-coded Doppler shift) can be obtained. Subsequently, according to the coding technique that was discussed by Goutelard [45] and was used to develop extremely low-power ionospheric sounders, Yao et al. [46] gave a detailed description of the operating principle in 2001, when the pseudorandom binary phase-coded pulse trains were adopted as the sounding waveform in WIOBSS. Moreover, the possibilities of applying some typical pseudorandom sequences into this waveform modulation were analyzed, such as the *m*-sequence and the Frank–Heimiller sequence. In practical operation, about 48 dB of additional signal processing gain can usually be obtained from pulse compression and coherent spectral integration to achieve ionospheric sounding with low power [47]. In 2005, the aforementioned operating principles were verified with practical experimental results by Zhao et al. [48]. At that time, the basic sounding principles for ionospheric observation were finally established. Though a phase method was put forward to estimate $f_{d}$ in a shorter detection time by Chen et al. [49] in 2007, this method has not been used in WIOBSS because it is difficult to distinguish the Doppler of several echoes arriving at the same time.

Since the binary phase-coded waveform is sensitive to the Doppler shifts, it cannot be applied in the case of fast-flying aircraft detection unless additional Doppler compensation algorithms are added. In 2014, Sun et al. [50] introduced the linear frequency-modulated continuous waveform (LFMCW) for WIOBSS. Due to the lower Doppler sensitivity of this waveform, it can be well employed for monitoring aircraft and the ocean. Furthermore, these classical sounding principles for observation of the aircraft and the ocean with LFMCW [51,52] have been added into WIOBSS.

In addition to the active detection, WIOBSS can also be configured as a passive radar to receive the signals emitted by existing HF broadcasts or non-cooperative HF radars to perform the SSL. According to Breit and Tuve’s theorem and Martyn’s equivalence path theorem, Li [53] described how to locate the longitude and latitude of the transmitter site when the elevation and azimuth angles of the received signal, the virtual reflection height, and the longitude and latitude of the receiver site are known.

### 2.2. System Configuration

Based on techniques of the integrated circuit chip, programmable digital signal processing, and field programmable gate arrays (FPGAs), Liu et al. [54] developed the initial version of WIOBSS in 2003 to validate the possibility of ionospheric backscatter sounding with an inter-pulse pseudorandom binary phase coded waveform. It consisted of a commercial HF receiver, a solid-state power amplifier, an AD9857 evaluation board from the Analog Device Corporation, a personal computer, two log-periodic antennas, a self-developed digital signal processing (DSP) board, and a transmit (T)/receive (R) switch module. The sounding parameters (e.g., the frequency points or frequency bands of interest, the pseudorandom sequences, and the operating parameters of the power amplifier and receiver) were all set in the operating interface with software [55]. After parameter setting, these parameters were sent to the AD9857 through the parallel port, to the DSP board through the peripheral component interconnect bus, and to the HF receiver via the IEEE 1394 bus. When the sounding time arrived, the FPGA on the DSP board produced the pseudorandom sequence and the timing control signals. The inter-pulse pseudorandom binary phase coded pulse trains were generated by a direct digital synthesizer (DDS) chip (AD9857) under the control of a personal computer and FPGA. By reprogramming the FPGA, the type and length of the pseudorandom sequence, the pulse width, and the duty cycle can be flexibly changed. During the initialization of whole system, the operating frequency, the link gain, and the parameters of digital downconversion (DDC) were set by a DSP in the receiver. After three frequency conversion processes in the analog front-end of the HF receiver, the intermediate frequency signal of 24 kHz was sampled by an analog-to-digital (A/D) converter with 16-bit resolution, and then digitally downconverted, decimated, and low-pass filtered in a dedicated DDC chip. Furthermore, these complex data streams with low sampling rates were sent from the HF receiver to the self-developed DSP board through the serial bus to perform pulse compression and coherent spectral integration. With this version of WIOBSS, two sounding modes can be achieved; that is, the fixed-frequency and the swept-frequency modes. In addition, the oblique backscatter ionogram and the figure of the scattering function can be obtained in the range of 1000 km with a power of about 50 W [56], but its performance was limited by some factors, as follows:

(1) Since a large number of cables and various data buses had been used, the electromagnetic compatibility and maneuverability were not desirable.
(2) The signal output from the AD9857 was only filtered by an 80 MHz low-pass filter, and was then sent directly to the power amplifier. As a result, the power of the output signal was very low, and its spectrum was not good because the harmonic and other unwanted signals were not filtered out.
(3) The reference clock of the whole system came from an Agilent E8491B module, and its phase noise was somewhat high.
(4) The receiver was not designed specifically for WIOBSS, and some of its parameters cannot meet the requirements well. Moreover, this receiver was dominated by the analogue circuits.

**Figure 1:**
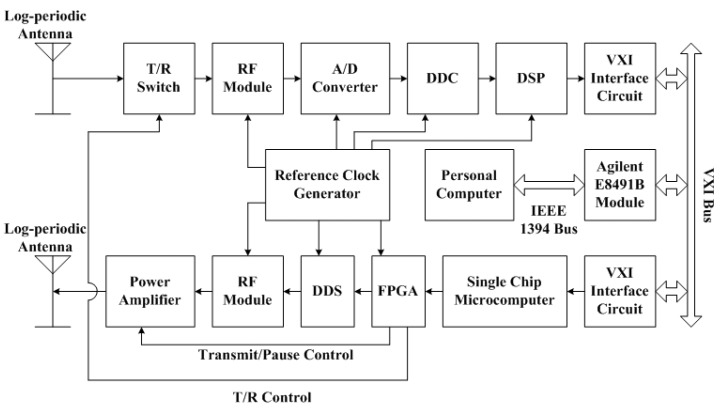
Block diagram of the second version of Wuhan Ionospheric Oblique Backscattering Sounding System (WIOBSS). A/D: analog-to-digital; DDC: digital downconversion; DDS: direct digital synthesizer; DSP: digital signal processor; FPGA: field programmable gate array; T/R: transmit/receive; VXI: Versa module eurocard eXtensions for Instrumentation.

In order to solve the aforementioned problems, the second version of WIOBSS was developed by Nie et al. [57] and Yao et al. [58] in 2005. Its block diagram is shown in Figure 1. This system mainly consisted of a personal computer, a transmitting channel, a digital receiver, two log-periodic antennas, a reference clock generator, and a solid-state power amplifier [59,60]. 

Compared with the initial version, several improvements were achieved in the second version. Firstly, all modules were designed in terms of the performance requirements of WIOBSS. Furthermore, based on the techniques of the virtual instrument bus and the modular design, the receiver, the transmitting channel, and the reference clock generator were designed as two Versa module eurocard eXtensions for Instrumentation (VXI) bus shielded *C* size (230 × 340 mm^2^) modules, and were inserted into the VXI bus mainframe for favorable electromagnetic compatibility and maneuverability. In practice, the personal computer communicated with the VXI mainframe via the IEEE 1394 bus, and the sounding parameters were sent to the two modules through the VXI bus. Secondly, the intermediate frequency was changed to be 1.4 MHz, and the twice-converted superheterodyne design was adopted in both the receiver and the transmitting channel to improve the spectrum and power of the output signal in the transmitting channel, and to reduce the analogue circuits in the receiver. Thirdly, the reference clock of all modules came from an oven-controlled crystal oscillator (OCXO) with high stability and low phase noise. Finally, integrated circuit chips having high performance were employed, such as the A/D converter, the DDC chip, and the DSP. Despite the aforementioned improvements, however, the phase of the output signal was seriously distorted by the narrow band-pass crystal filter, resulting in a large error in the Doppler frequency estimation. In addition, the implementation of the VXI interface protocol was very complicated, and the cost of the system based on the VXI bus was slightly high.

**Figure 2:**
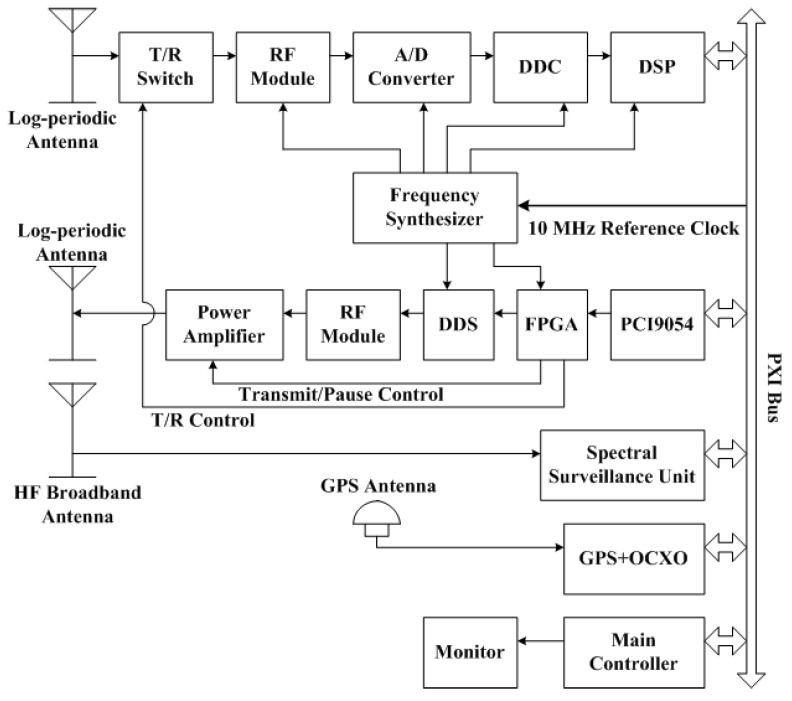
Block diagram of the third version of WIOBSS. GPS: Global Positioning System; HF: high-frequency; OCXO: oven-controlled crystal oscillator; PXI: Peripheral component interconnect eXtensions for Instruments.

In 2009, the third version of WIOBSS was successfully developed by Yang et al. [61,62,63]. On the one hand, the time and frequency synchronous controller in which a high-performance OCXO is disciplined by the Global Positioning System (GPS) was designed by Shi et al. [64]. With this module, WIOBSS can not only be configured as a bistatic radar to achieve the oblique-incidence sounding at the time of the oblique backscatter sounding [65,66], but can also be configured as a multistatic radar to detect the target in a much larger surveillance area and to extract more information from the target [67], such as the ionospheric oblique-incidence sounding network [68]. On the other hand, according to the specification of the Peripheral component interconnect eXtensions for Instruments (PXI) bus (which has been reported as high-performance and functional, reliable, easy to integrate and use, cost-effective, and portable [69]), the system was designed as a modular and compact PXI-based system. As shown in Figure 2, it consisted of a main controller, a transmitting channel, a digital receiver, antenna systems, a synchronous controller, a spectral surveillance unit, and a solid-state power amplifier. Except for the antenna systems and the power amplifier, the whole system was designed as one PXI bus shielded 6U-sized (233.35 × 160 mm^2^) module and five PXI bus shielded 3U-sized (100 × 160 mm^2^) modules, and its dimensions were 177.8 mm × 431.8 mm × 457.2 mm. All modules were inserted into a PXI bus mainframe PXI-1056 from National Instruments Corporation. In order to reduce the external cable connections, the trigger bus and the local bus distributed in the PXI backplane were used to transfer the analog signals and the high-speed digital signals among modules. Furthermore, an embedded main controller PXI-8106 running in Windows XP was adopted as the control center to replace the personal computer, and a spectral surveillance unit was used to measure the background external noise spectral density and to monitor the channel occupancy of the HF band. In addition, the sounding waveform generated by the AD9857 was first filtered by a 3–30 MHz band-pass filter, then amplified by a low-noise amplifier and filtered by the eight-band sub-octave filters, and finally sent to the power amplifier. With this design, the generated waveform can be an arbitrarily designed waveform, and the harmonic and other unwanted signals can be removed well without distorting the phase of the emitted signal. Since the operating principle of the vertical-incidence sounding is very similar to that of the oblique backscatter sounding, Yao et al. used the intra-pulse pseudorandom binary phase-coding technique to achieve the function of vertical-incidence sounding for the third version of WIOBSS in 2010 [70]. Using this system, the oblique backscatter ionogram and Doppler ionogram can be obtained in the range of 2500 km with a power of about 200 W, and the vertical- and oblique-incidence sounding results can be clearly obtained with a power of no more than 100 W [71]. Moreover, a new sounding mode (i.e., the frequency hopping mode) was added to acquire the ionospheric state over several frequency bands of interest, to avoid radio frequency interference, and to reduce the cause of interference to other HF systems [72].

**Figure 3:**
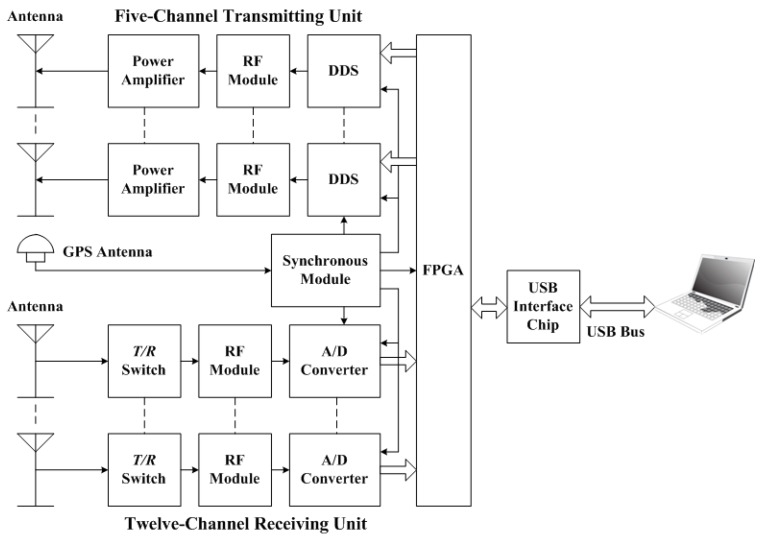
Block diagram of the latest version of WIOBSS.

**Figure 4:**
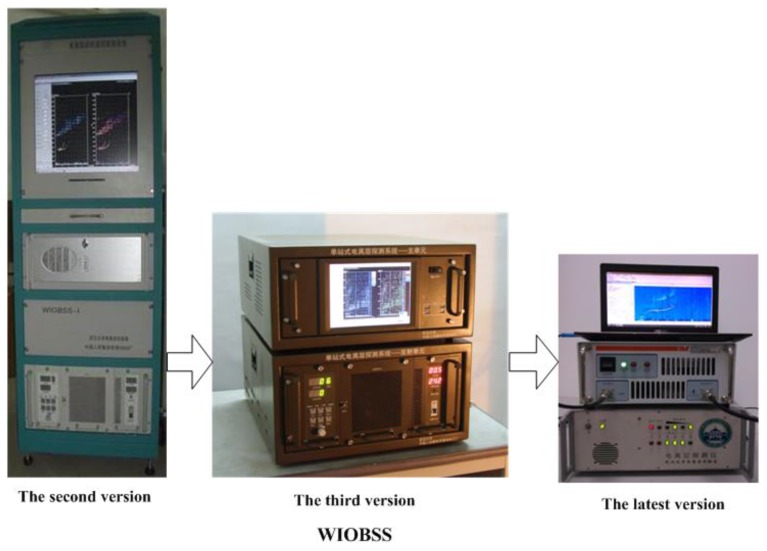
Evolution of WIOBSS over three decades.

From 2011, a novel portable and low-power WIOBSS having multiple digital channels and more applications began development. In 2013, Huang et al. [73] used the Universal Serial Bus (USB) as the system bus to reduce the cost and the dimensionsof WIOBSS. Furthermore, the DDC was implemented with the intellectual property core in a high-performance FPGA which is low-cost and convenient to modify. In order to further reduce the system cost and size, and to achieve higher flexibility, the intermediate frequency of 41.4 MHz was obtained with just one frequency conversion in the analog front-end, and was digitalized in terms of the band-pass sampling theory. According to the concept of software radar [74], the pulse compression and the coherent spectral integration were performed in a laptop instead of the DSP. In addition, a four-channel digital receiver was designed to measure the two-dimension direction of arrival (DOA) of the signals emitted by existing HF broadcasts. The azimuth angle can be accurately measured with this system, but a large error existed in the elevation angle measurement, partly due to the limited number of the receiving channels [75]. Therefore, a twelve-channel digital receiver was developed by Sun [76] in 2015 to improve the measurement accuracy of the two-dimension DOA and to perform the SSL. At the same time, a five-channel transmitting unit was developed by Cui et al. [77] to form multiple beams or to simultaneously generate different sounding waveforms at multiple frequencies. It had been used to obtain the ionospheric electron density information over a larger area and to achieve the ocean sensing via the hybrid sky–surface wave mode. Since the VHF scattering signals are very useful for detecting the natural and artificial ionospheric irregularities [78], the operating frequency band of WIOBSS was further extended to VHF by Sun et al. [79]. Finally, some integrated circuit chips having higher performance and lower power dissipation were employed in this system, such as the AD9911 DDS chip and the LTC2203 A/D converter. The block diagram of the latest version of WIOBSS is shown in Figure 3, integrating aforementioned improvements. It can be seen that this system consists of a five-channel transmitting unit, a twelve-channel receiving unit used for the reception of HF and VHF echoes, a synchronous module, antenna systems, power amplifiers, and a laptop. Compared with the original versions of WIOBSS, the dimension of this multichannel radar becomes smaller (as shown in Figure 4), and it has a higher degree of flexibility for multifrequency and multimode operations, broader operating band, and more applications. Furthermore, this radar can be built at a low cost and is easy to install to make operations at many sites around our country feasible.

### 2.3. Sounding Waveforms

It is well known that the waveform design is very important for radar performance, and is influenced by the radar configuration and the target characteristic. Up to now, four types of sounding waveforms have been employed in WIOBSS.

**Figure 5:**
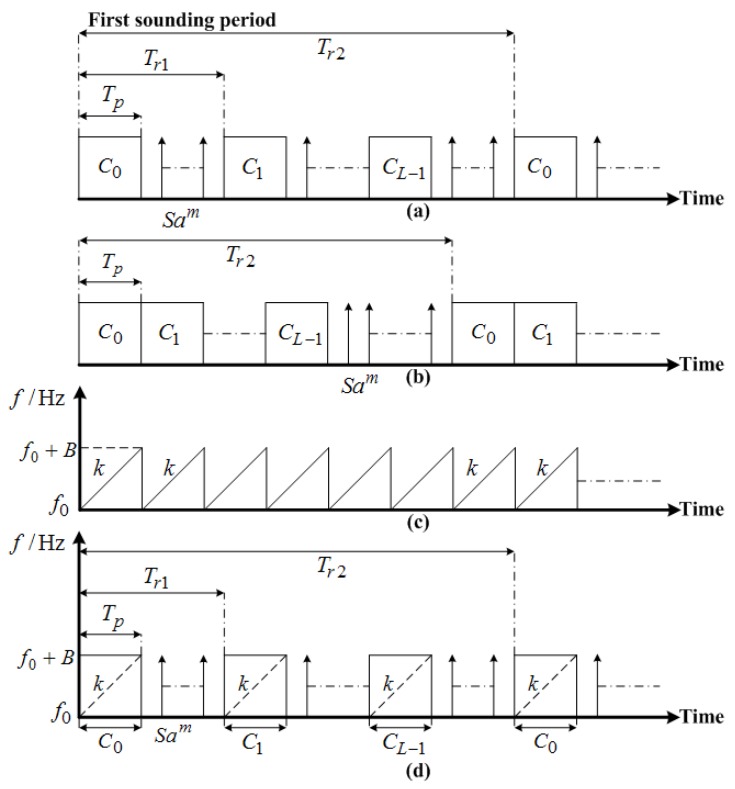
Sounding timing of the four types of waveforms employed in WIOBSS. (**a**) The inter-pulse binary phase-coded pulse trains. (**b**) The intra-pulse binary phase-coded waveform. (**c**) The linear frequency-modulated continuous waveform (LFMCW). (**d**) The inter-pulse pseudo-random binary phase-coding waveform where the sub-pulse uses the chirp modulation. $T_{p}$ represents the pulse width, L denotes the length of the coding sequence, $T_{r1}$ denotes the pulse repetition period, $T_{r2}$ denotes the sequence repetition period, $C_{l}$ is the element of {+1, −1} in a pseudo-random coding sequence, l=0,⋯,L−1, $Sa^{m}$ represents the received echo at the *m*th range bin, k is the chirp rate, $f_{0}$ represents the initial center frequency of the chirp signal, and B is the sweep bandwidth of the chirp signal.

The first type is the inter-pulse binary phase-coded waveform that is usually used for the oblique backscatter sounding and the parasitic oblique-incidence sounding. When WIOBSS is configured as a monostatic radar, the inter-pulse binary phase-coded pulse trains should be adopted. On the other hand, when WIOBSS is configured as a quasi-monostatic radar to avoid the strong interference imposed by the leakage of the transmit power and to increase the average transmit power, the binary phase-coded continuous waveform is employed. In the first case, it can be seen from Figure 5a that the coded pulses are transmitted with equal pulse interval, and the intervals between two adjacent pulses are used for reception. Hence, the small blind zones are evenly distributed in the group distance, and can be reduced with the technique of diverse pulse repetition intervals proposed by Yao et al. [80]. In the latter case, the coded waveform is continuously transmitted in the transmitting site, and the echoes are continuously received in the receiving site. Therefore, no blind zone exists in the range coverage. In addition, very long pseudorandom sequences are adopted in both cases to achieve a high signal processing gain, such as the *m*-sequences and the almost perfect autocorrelation sequences [81,82], and are circularly transmitted to fulfill its good periodic autocorrelation function (ACF) [83]. In terms of the ambiguity function of this waveform, its characteristic parameters are shown in Table 1. Though this waveform is sensitive to the Doppler shifts, it can be well applied in the ionospheric sounding because the Doppler shift of the ionosphere does not exceed ±2 Hz at mid-latitude sites in most cases [67]. In practice, a sidelobe suppression filter is usually added to reduce the range sidelobe and to decrease the peak sidelobe level.

The second type is the intra-pulse binary phase-coded waveform that is always used for the vertical-incidence sounding. As shown in Figure 5b, the coded pulses are first transmitted, and then the echoes are continuously received. Therefore, a large blind zone exists in the group distance. However, this is not a problem for the vertical-incidence sounding, because the lowest height of the ionosphere that can be observed by the ionosonde is about 90 km. Furthermore, this waveform requires that the coding sequence should have an “ideal” aperiodic ACF. Some sequences (e.g., the Barker sequence and the complementary code) possess such ideal aperiodic ACF property, and hence can be employed [83]. Though the length of such pseudorandom sequences is short, resulting in a slightly low signal processing gain obtained from the pulse compression, the final signal-to-noise ratio is still considerable due to the short distance of the target in the vertical-incidence sounding. In practical operation, the complementary code is usually chosen to modulate the phase of the sounding waveform because its range peak sidelobe level is equal to 0 dB [84]. Furthermore, the sounding waveforms which are coded by the complementary sequence pairs should be emitted in turn by the antenna systems. The characteristic parameters of the intra-pulse binary phase-coded waveform in which the complementary code is used are shown in Table 1.

The third type is the LFMCW. Different from the aforementioned binary phase-coded waveforms, LFMCW is easy to generate and is not sensitive to the Doppler shifts [85]. Therefore, it can be well applied for aircraft detection and ocean sensing. Furthermore, when LFMCW is adopted (as shown in Figure 5c), the group distance without a blind zone and high average transmit power can be achieved in WIOBSS due to the bistatic or quasi-monostatic configuration. In addition, by adding different initial phase in each linear frequency sweep and then transmitting them at different carrier frequencies, multiple-input multiple-output (MIMO) radar waveforms can be obtained and have been used in WIOBSS to perform ocean sensing via the hybrid sky–surface wave mode. The characteristic parameters of LFMCW are shown in Table 1. In practice, a window function should be added to reduce the range peak sidelobe level, and the range-Doppler cross-coupling should be considered for the detection of fast-flying targets.

The last type is the sounding waveform, combining pseudorandom binary phase-coding and chirp modulation. The remarkable characteristic of this waveform is that it possesses not only the virtues of the binary phase-coded signal, but also the advantages of the chirp signal. Compared with the binary phase-coded waveform, higher range resolution and larger signal processing gain can be achieved with this waveform. Furthermore, it is more insensitive to large Doppler shift. On the other hand, this waveform can also achieve larger signal processing gain than the LFMCW, and it is not subjective to significant range-Doppler cross-coupling. Therefore, this waveform can have more applications than the binary phase-coded waveform and the LFMCW—especially for the low-power HF radar used to detect fast-flying targets. Since this waveform has four subclasses—i.e., the inter-pulse pseudo-random binary phase-coding waveform where the sub-pulse uses the chirp modulation, the inter-pulse pseudo-random binary phase-coding waveform where the whole coding sequence uses the chirp modulation, the intra-pulse pseudo-random binary phase-coding waveform where the sub-pulse uses the chirp modulation, and the intra-pulse pseudo-random binary phase-coding waveform where the whole coding sequence uses the chirp modulation—Shi et al. [86] analyzed the feasibility of applying them in WIOBSS. The sounding timing of the inter-pulse pseudo-random binary phase-coding waveform where the sub-pulse uses the chirp modulation is shown in Figure 5d, and its characteristic parameters are shown in Table 1. Furthermore, it has been applied in WIOBSS for ionospheric oblique backscatter sounding and aircraft detection [87]. In practical operation of this waveform, a sidelobe suppression filter should be added to reduce the range peak sidelobe introduced by both the binary phase-coded signal and the LFMCW.

### 2.4. Signal Processing

When WIOBSS has just one receiving channel, its signal processing mainly consists of pulse compression, coherent spectrum integration, echo signal detection, interference suppression, and the calibration of systematic delays. The pulse compression and coherent spectrum integration were described in detail in [46,76] when the binary phase-coded waveform and the LFMCW were adopted, respectively. Furthermore, Chen [88] introduced the constant false alarm rate (CFAR) algorithm for echo signal detection in WIOBSS, and Wang [89] developed the adaptive CFAR processor to increase the probability of detecting weak echoes. Since the systematic delays can produce errors in the group distance measurement, Fang et al. [90] used the near-field signal to calibrate the systematic delays. On the other hand, as described by Reinisch et al. [91], the HF receiver having a large bandwidth is susceptible to wideband background noise and narrow-band interferences. The background noise can be effectively mitigated with methods such as signal integration, pulse modulation, and increased transmission power. However, it is difficult to remove the narrow-band interferences which come from other HF transmitters with unpredictable occurrences at a priori unknown frequencies. In order to largely suppress these narrow-band interferences in WIOBSS, many signal processing algorithms were proposed, such as CFAR (based on the statistical feature of echoes [90]), the radio-frequency interference mitigation method proposed by Chen et al. [92], and the second-order statistics filter proposed by Wang et al. [93]. In addition, some sidelobe suppression filters are introduced after the pulse compression, such as the Hamming window [87] and the range sidelobe filter described in [94].

When WIOBSS has multiple transmitting and receiving channels, apart from the aforementioned signal processing modules, channel calibration, digital beam forming, and the estimation of DOA should also be included. In order to eliminate the amplitude and phase differences among the transmitting or receiving channels, an algorithm based on eigenvalue decomposition was proposed by Cui et al. for the channel calibration [77]. Moreover, the digital beam forming is implemented by inserting a phase delay in the DDS of each transmitting channel, and this phase delay is varied with frequency to produce the equivalent beams at different sounding frequencies [95]. In addition, the multiple signal classification algorithm [96] is adopted to estimate the two-dimensional DOA of various echoes.

In practical applications, when WIOBSS is used for aircraft detection and sea-state sensing, the methods described in [97] were adopted for decontamination of the ionosphere distortion by Chen [88]; these include short coherent integration time, phase gradient, and eigen-decomposition. Furthermore, a method based on neural networks and time-frequency representation was proposed for aircraft detection by Li et al. [98]. Since the multipath propagation can severely degrade the detection ability of WIOBSS when it is used for aircraft detection, sea-state sensing, and SSL, the Viterbi algorithm based on the hidden Markov model was proposed by Zhou et al. [99] to perform the mode recognition, and then to eliminate the influence of multipath propagation. 

### 2.5. Data Processing

When WIOBSS is used for ionospheric observation, the data processing is mainly focused on trace extraction, automatic scaling, and electron density profile inversion from various ionograms. With respect to the vertical-incidence ionograms, an algorithm using Kalman filtering was proposed by Su et al. to form traces and separate the ordinary trace and the extraordinary trace [100]. Subsequently, according to the ionosphere model named “quasi-parabolic segments”, Jiang et al. proposed several methods for the automatic calculation of electron density profiles from various types of vertical-incidence ionograms [101,102,103,104,105]. Furthermore, a software named ionoScaler was developed by Jiang et al. to carry out manual and automatic scaling of vertical-incidence ionograms [106]. With this software, not only the critical frequency and the virtual height of *E* layer, sporadic *E* layer, *F*1 layer, and *F*2 layer, but also the vertical total electron content (TEC) can be automatically obtained from the vertical-incidence ionograms—even during periods of geomagnetic storms. As for the processing of oblique-incidence ionograms, an inversion technique with which the oblique-incidence ionograms are converted to vertical-incidence ionograms to achieve the critical frequency, the minimum virtual height, and the electron density profile of the *F* layer was proposed in terms of Martyn’s equivalent path theorem and the international reference ionosphere (IRI) model by Chen et al. [107]. Using the hybrid genetic algorithm and the quasi-parabolic model, an automatic scaling method was proposed by Song et al. to achieve the maximum usable frequency and the group path of various ionospheric layers from the oblique-incidence ionograms [108,109,110,111]. Different from the vertical- and oblique-incidence ionograms, the swept-frequency backscatter ionograms contain the information concerning the ionospheric condition as well as the scattering characteristic of the surface of the Earth. Moreover, a full inversion to get the underlying ionospheric structure is extremely difficult [112], especially for the backscatter ionograms obtained by WIOBSS with a low transmit power. Therefore, our works concentrate on the leading edge of the swept-frequency backscatter ionograms. Through the use of minimum group path delay theory, an automatic scaling algorithm of the swept-frequency backscatter ionograms was proposed by Song et al. [113]. Moreover, many algorithms have been proposed to invert the leading edge of the swept-frequency backscatter ionograms to achieve the electron density profile, such as the inversion algorithm using simulated annealing [114], the ionospheric *F*2 region inversion approach utilizing a single quasi-parabolic ionospheric model [115], or the IRI model combined with ray-tracing techniques [116]. In order to validate these inversion algorithms, several experiments were conducted by Zhou et al. to make comparisons between the ionospheric electron density distributions reconstructed by GPS computerized tomography, backscatter ionograms, and vertical ionograms [117]. 

In addition to the processing of ionograms, the control platform operating software is also a significant component in the data processing when many sounders are placed over a large region to build a sounding network. Similar to the technical solutions provided for the Digisonde portable sounder [118], operating software was developed with techniques including virtual private networking and database management by Jiang [119] to achieve the routine unattended operations for WIOBSS. 

When WIOBSS is used for aircraft detection and sea-state sensing, its coverage under various ionospheric conditions and different beam directions of the antenna is important for target observation. An inversion algorithm with which the ground range on the leading edge of the backscatter ionograms can be obtained without electron density was proposed by Su et al. [120]. Furthermore, the coverage of WIOBSS displayed on a map-based user interface was developed by Li et al. [121]. In addition, as described in [122], coordinate registration is one of the most critical techniques in any HF skywave radar. Hence, Zhou et al. analyzed the effects of the travelling ionospheric disturbance and the ionospheric changes on the coordinate registration [123,124]. Subsequently, Song et al. further proposed a method based on Bowring’s equation for target localization [99,125].

## 3. Remote Sensing Applications of WIOBSS Operating at Low Transmit Power

As mentioned before, WIOBSS was initially designed to be a low-power and small-size HF backscatter radar to survey a large region of the ionosphere in real-time. With the introduction of the advanced radar technology, other applications have also been achieved with this radar, such as ionospheric research using vertical- and oblique-incidence soundings, earthquake research, HF channel evaluation, SSL, aircraft detection, and sea-state sensing. A brief review of its various remote sensing applications is given below to demonstrate the good performance of WIOBSS after its new system design.

### 3.1. Ionospheric Research

When a power of no more than 150 W is used for transmission, typical vertical-incidence, oblique-incidence, and oblique backscatter ionograms obtained with WIOBSS were given in [126]. Through the use of the aforementioned data processing methods, the critical frequency and the virtual height of various ionospheric layers, the electron density profile, and the TEC can be achieved from these ionograms to study the dynamical processes in the ionosphere and the underlying driving mechanisms. In terms of the vertical-incidence ionograms obtained in the low-latitude and equatorial regions, Jiang et al. analyzed the spread *F*, the *F*2 layer stratification, and their formation mechanisms [127,128,129]. On the other hand, Zhang et al. investigated the scales and drift speeds of low-latitude ionospheric field-aligned irregularities with the oblique backscatter ionograms [130]. Based on the multistatic backscatter ionograms and the parasitic oblique-incidence ionograms, Shi et al. described the calculation method and the experimental results for localization of the ionospheric field-aligned irregularities [66]. Zhou et al. analyzed the characteristics and formation mechanisms of the low-latitude daytime large-scale traveling ionospheric disturbances during a geomagnetically quiet period [131]. Gong et al. studied the scale distribution of the field-aligned irregularities in the *E*-region [132]. During the solar eclipse of 22 July 2009 in Wuhan, in terms of the multistatic sounding results provided by WIOBSS, Zhang et al. found that both the sporadic *E* and *F* layers were influenced by gravity waves generated in the atmosphere during the solar eclipse [133]; Chen et al. analyzed the solar eclipse’s effects on the sporadic *E* layer [134,135,136,137]. For the solar eclipse on 15 January 2010, Chen et al. used the vertical-incidence and oblique-incidence sounding results to analyze the solar eclipse effects on the *F*2 layer [138,139]. Note that ionospheric variations may affect the performance of other HF radar systems [140]; therefore, ionospheric research is very important for HF radar applications.

### 3.2. Earthquake Research

Due to the seismo-ionospheric anomalies which appear either a few days to weeks before large earthquakes or around the earthquake time [141], many researchers have used ground-based vertical-incidence sounders [142], GPS TEC observations [143], and topside vertical soundings from satellites [144] to study the variations in the ionosphere over seismically active regions several days or hours before strong earthquakes. In 2009, Zhao et al. analyzed the possibility of applying multifunctional WIOBSS into the ionospheric observation before and after the earthquake [145]. In 2016, Liu et al. used the vertical-incidence ionograms provided by WIOBSS to analyze the ionospheric disturbances before the Nepal earthquake [146].

### 3.3. HF Channel Evaluation

Due to the significant advantages of HF communication, such as long-distance action, high mobility, low cost, and the ability to provide communication in times of natural disasters, the HF channel still attracts a lot of attention [147,148]. With the oblique-incidence ionograms provided by WIOBSS, several parameters of the HF channel can be obtained in real-time, including the lowest usable frequency, the maximum usable frequency, the Doppler spread, the multipath spread, the attenuation, and the signal-to-noise ratio. Furthermore, Zhou et al. established a real-time mapping model of the critical frequency of the *F*2 layer in Northern China by using neural networks [149], and investigated the ionospheric HF channel reciprocity at middle latitudes [150].

### 3.4. SSL

As a powerful tool to find the position of an HF transmitter or a signal source, SSL has some remarkable advantages when compared with the multi-site location techniques, including the requirement for less equipment, lower cost, and more convenient installation. Hence, it has found applications in search and rescue, enforcement of frequency regulations, and in military situations [151]. Generally speaking, there are three design components for developing an SSL system; i.e., the SSL technique, the signal direction information, and the source of ionosphere data [152]. Based on the relationships of the secant law, Breit and Tuve’s theorem, and Martyn’s equivalence path theorem, Li described the geolocation method and the direction-finding algorithms when WIOBSS is applied in SSL [53]. As mentioned before, many inversion algorithms had been developed to obtain the electron density profile from various ionograms provided by WIOBSS, and then the virtual height can be derived by finding the reflection point which is estimated with both the intensity and the spatial distribution of the electron density profile. According to this technical solution, Zhu et al. proposed an SSL system using the technique of ionospheric backscatter sounding, and most of the errors of the geo-locations are within 10% [153].

### 3.5. Sea-State Sensing

Different from the traditional sea-state sensing using skywave or surface-wave HF radars, the HF hybrid sky-surface wave radar uses a new propagation mode composed of a skywave transmit path and a surface wave receive path [154]. With this new detection technique, the targeting over-the-horizon detection and the ocean surface dynamical environment surveillance capability can be improved [155]. Several experiments were conducted when WIOBSS was configured as an HF hybrid sky-surface wave radar to determine the sea state. During these experiments, the oblique-incidence sounding was first adopted to choose the optimum operating frequency, and then the Doppler spectrum of the echoes from the ocean was obtained with the LFMCW [126,156]. This is similar to the “listen before talk” procedure in HF radar applications [157]. 

### 3.6. Aircraft Detection

A large antenna array and high transmit power are usually needed for the HF skywave over-the-horizon radar to detect aircraft [3]. However, WIOBSS does not possess such conditions because of its characteristics of low transmit power and small size. Therefore, this radar is configured as a bistatic radar to record the echoes scattered directly from the aircraft. Several experiments were performed when the fixed-frequency sounding mode was adopted [67,98,158]. As described in [87], the aircraft can be successfully detected with WIOBSS by using the method of time-frequency representation. 

## 4. Conclusions and Perspective

Although some good sounding results have been achieved by WIOBSS after its new system design, the performance of this radar is still limited by many factors, such as the lack of a large antenna array and the influence of multipath propagation. On the other hand, some new radar technology, including MIMO radar technology and satellite-based sounding technology, have not been applied in WIOBSS. According to these problems existing in this radar, several recommendations and potential solutions are given as follows to further improve its capabilities.

### 4.1. Digital Array Radar Technology

Digital array radar (DAR) is a fully-digitized phased array radar in which digital beam forming techniques are used both in reception and transmission [159], and this technology has been successfully applied in HF radar, such as the SuperDARN HF radars [160]. If DAR technology is used in WIOBSS, the two-dimensional angle-of-arrival of echoes can be accurately measured to study the ionospheric irregularities, the fine structure of the ionosphere, and so on. Furthermore, the narrow-band interference can be effectively suppressed with the adaptive null-forming scheme, and multiple transmit beams can be formed simultaneously to detect the target over a vast region. On the other hand, the interference produced by our radar can also be effectively reduced in the spatial domain. Despite the above advantages offered by the DAR technology, several technical challenges should be overcome before this technology is used in WIOBSS. For example, the swept-frequency sounding mode is always used in WIOBSS to gain an overall appraisal of propagation conditions as a function of range, azimuth, and operating frequency over a vast area from a single location. However, this wideband operation can introduce several problems for the antenna array design because the optimum spacing between antennas, the side lobe, and the grating lobe change with operating frequencies [161]. Moreover, the phase calibration and the 2π phase ambiguity should also be investigated.

### 4.2. MIMO Radar Technology

Motivated by developments in MIMO communication systems, MIMO technology is introduced for radar to overcome the phenomenon of degradation which is caused by RCS fluctuations [162], to improve the spatial spectral resolution [163], to restrain strong interference [164], and so on. Furthermore, this technology can be broadly categorized into four areas: spatial diversity radar, localization using multi-lateration radar, high spatial resolution radar using regular sparse arrays, and non-causal radar-transmit beamforming [165]. The last application area has attracted a great deal of attention from researchers in the field of HF skywave over-the-horizon radar because it can be used to reject the spatially-discrete Doppler-spread clutter [166].

In recent years, WIOBSS have been configured as the MIMO radar to achieve the localization of ionospheric irregularities [66] and to enhance the detectability of targets [67]. In the future, non-causal beamforming implemented with the MIMO radar architecture should be considered to remove the influence of the ionospheric multipath propagation when WIOBSS is used for aircraft detection and sea-state sensing and, on the other hand, to study the multi-layered structure of the ionosphere in real-time and the propagation characteristics of HF signals in such multi-layered media. Furthermore, several critical problems should be solved before the non-causal beamforming is applied in WIOBSS, such as the orthogonal waveform design, the analysis of the effects of ionospheric propagation on the orthogonality of the sounding waveform, the optimal design of two-dimensional transmitting and receiving antenna arrays, and the selection of appropriate adaptive algorithms [167,168].

### 4.3. Ionospheric Joint Observations with Different Sounding Techniques

As described by Reinisch [169], each ionospheric sounding technique has its own objectives and can only provide limited information about the ionospheric state. Due to the complex nature of these phenomena in the ionosphere, it is very important to perform the ionospheric observations with different sounding techniques simultaneously to better understand the underlying physical mechanisms [29,170]. In addition to WIOBSS, which can be used for vertical-incidence sounding, oblique-incidence sounding, and coherent HF scatter probing, other different sounders have also been developed by the Ionospheric Laboratory of Wuhan University, such as the ground-based extremely low frequency (ELF)/very low frequency (VLF) receiver [171], the HF Doppler sounder, the Wuhan mesosphere–stratosphere–troposphere radar operating in the VHF band [172], and the GPS receiver used for the TEC measurements. Furthermore, a meteor radar used for the observation of neutral winds is also under development. Therefore, when WIOBSS is used for the ionospheric observation, the aforementioned sounding techniques should also be used to jointly observe the ionosphere to provide an improved understanding of the ionospheric processes and the underlying driving mechanisms in the mid-latitude and low-latitude regions. 

### 4.4. Passive Radar Technology

Passive radar systems use the illuminators of opportunity instead of a dedicated radar transmitter as the signal source. These systems offer many advantages over conventional monostatic radars, including system cost reduction, smaller size, being more difficult to detect and hence less susceptible to electronic countermeasures, and the ability to detect the reduced RCS targets [173,174]. As for WIOBSS, passive radar technology can be employed to detect the aircraft target and the ionosphere at several fixed frequencies when the HF/VHF broadcast signals are used as the sounding signal. For example, the VHF broadcast signals can be passively received to study ionospheric irregularities [175,176]. In addition, a number of technical challenges need to be overcome before passive radar technology can be used in WIOBSS, such as waveform analysis in the bistatic geometry, the effective removal of unwanted multipath components and co-channel interference coming from other sources, and the range and Doppler spectrum estimation. 

### 4.5. Spaceborne Ionospheric Sounding Technology

Different from the ground-based ionospheric sounder, the ionosonde carried by the satellite or spacecraft can provide valuable information for revealing the structure of the high ionosphere and for investigating the resonance properties of a magnetoplasma, such as obtaining the electron density profiles on a global scale [177]. Furthermore, it is the only means to observe the ionosphere of other planets, such as the radar soundings of the ionosphere of Mars [178]. The rapid development of microsatellites and minisatellites has also been providing an opportunity for the spaceborne ionospheric sounding technology because of their low cost and rapid response [179]. In 2010, Yao had described the research of the spaceborne multi-function plasma sounding system [180]. In the future, we will improve the system configuration of WIOBSS to make it suitable for being carried by the minisatellite or spacecraft, and a number of technical challenges need to be overcome before the spaceborne WIOBSS can be turned into an operational system, such as the reduction of the HF antenna size, the design of the data storage and transmission system, and the development of small-size and low-power components.

## Figures and Tables

**Table 1:** Waveform characteristic parameters.

Item	Inter-Pulse Binary Phase-Coded Waveform	Intra-Pulse Binary Phase-Coded Waveform	LFMCW	Combined Waveform
Unambiguous delay detection range	$T_{r2}$	$T_{r2}$	$T_{p}$	$T_{r2}$
Delay resolution	$T_{p}$	$T_{p}$	$1/(kT_{p})$	$1/(kT_{p})$
Unambiguous Doppler detection range	$1/T_{r1}$	$1/T_{r2}$	/	$1/T_{r1}$
Doppler resolution	$1/(NT_{r2})$ ^1^	$1/(NT_{r2})$	$1/(MT_{p})$ ^2^	$1/(NT_{r2})$
Ideal pulse compression gain(dB)	20log_10_(*L*)	20log_10_(*L*)	20log_10_(*BT_p_*)	20log_10_(*BT_p_*)+20log_10_(*L*)
Blind zones	$cT_{p}$ ^3^	$cLT_{P}$	/	$cT_{p}$ ^3^

^1^ N denotes the number of a circularly transmitted sequence. ^2^ M denotes the number of the chirp signal. ^3^ Blind zones are distributed with an equal interval of $c(T_{r1}-T_{p})$, where c is the speed of light.
